# Malaria-Infected Mice Are Cured by a Single Low Dose of a New Silylamide Trioxane Plus Mefloquine

**DOI:** 10.3390/ph2030228

**Published:** 2009-12-21

**Authors:** Lauren E. Woodard, Bryan T. Mott, Vandana Singhal, Nirbhay Kumar, Theresa A. Shapiro, Gary H. Posner

**Affiliations:** 1 Department of Chemistry, School of Arts and Sciences, The Johns Hopkins University, 3400 North Charles Street, Baltimore, Maryland 21218-2685, MD, USA; Emails: lwoodar1@jhu.edu (L.E.W.); bmott2@jhu.edu (B.T.M.); 2 Department of Molecular Microbiology and Immunology, Malaria Research Institute, Johns Hopkins Bloomberg School of Public Health, Baltimore, MD Baltimore, MD 21205, USA; Emails: vsinghal@jhsph.edu (V.S.); nkumar@jhsph.edu (N.K.); 3 Division of Clinical Pharmacology, Department of Medicine, School of Medicine, The Johns Hopkins University, Baltimore, MD 21205, USA; Email: tshapiro@jhmi.edu (T.A.S.)

**Keywords:** silylamide trioxanes, antimalarial monomers and dimmers, single dose oral cure

## Abstract

Three thermally and hydrolytically stable silylamide trioxanes have been prepared from the natural trioxane artemisinin in only five simple chemical steps and in at least 56% overall yield. Two of these new chemical entities completely cured malaria-infected mice at a single oral dose of only 8 mg/kg combined with 24 mg/kg of mefloquine hydrochloride. The high efficacy of this ACT chemotherapy is considerably better than the efficacy using the popular trioxane drug artemether plus mefloquine hydrochloride.

## 1. Introduction

Malaria is one of the world’s most widespread infectious diseases [1]. Much effort is currently being devoted to develop effective vaccines to prevent people from becoming infected with malaria parasites [2,3]. Chemotherapy of people having malaria using such antimalarial amines as chloroquine has been effective for over 50 years [1]. The increasingly widespread resistance of malaria parasites to chloroquine and related antimalarial amines [4], however, has stimulated a search for new natural and synthetic antimalarials. 

Progress in chemotherapy has been made using protease inhibitors to starve the parasites [5,6], using antimalarial acridones [7] and new 4-aminoquinolines [8] to counteract resistance, and using some modified chloroquine analogs [9]. Trioxanes derived from artemisinin (1), such as artemether (**2c**) and sodium artesunate (**2d**), are now being used clinically to cure malaria-infected people [10,11,12,13,14,15,16]. The World Health Organization (WHO) has recommended that a combination of a trioxane plus an established amino antimalarial should be used chemotherapeutically [17]. Artemisinin combination therapy (ACT) usually involves 3-6 doses of a trioxane plus an amino antimalarial administered to a malaria-infected patient over several days [18,19,20,21,22]. Patient compliance would be improved and cost lowered by a single dose oral cure. Toward this important medical goal, we have reported cure of malaria-infected mice by a single subcutaneous dose of a trioxane dimer [23], a related series of trioxane dimers curative after three oral doses [24], a trioxane dimer sulfone plus mefloquine curative after only a single oral dose [25], a fluorinated amide trioxane monomer plus mefloquine curative after only a single oral dose [26], and also a 5-carbon-linked trioxane dimer plus mefloquine curative after only a single oral dose [27].

**Figure 1:**
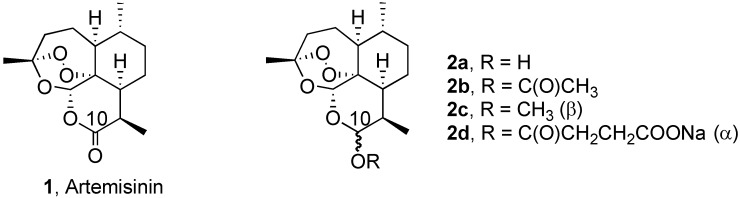
First Generation Semi-Synthetic Derivatives of Artemisinin.

Only recently has the beneficial effect of introducing a silicon atom in place of a carbon atom in some clinically used drugs begun to be appreciated [28]. This new appreciation inspired us to synthesize some silicon-containing trioxanes and to determine their antimalarial efficacy in rodents.

**Scheme 1:**
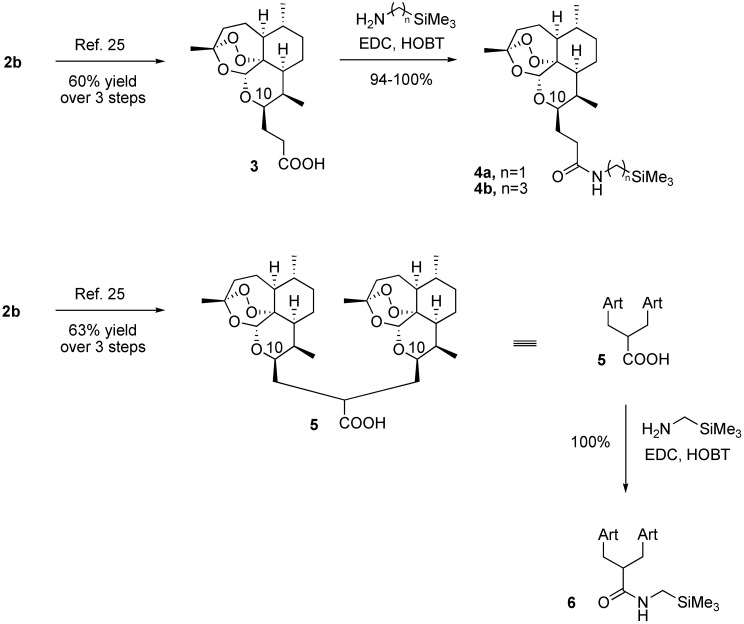
Monomer and Dimer Silylamide Derivatives of Artemisinin.

We report here an easily synthesized, new family of silylamide trioxane monomers **4** and dimer **6** (Scheme 1) able to cure malaria-infected mice after only a single 8 mg/kg oral dose combined with 24 mg/kg of mefloquine hydrochloride. 

## 2. Results and Discussion

### 2.1. Chemistry

Chemical synthesis of new silylamide trioxane monomers **4a** and **4b** and silylamide trioxane dimer **6** from the natural artemisinin (**1**) proceeded easily on gram scale in at least 56% overall yield via dihydroartemisinin acetate (**2b**) and then via either trioxane monomer carboxylic acid **3** or trioxane dimer carboxylic acid **5** (Scheme 1). Quantitative amide formation was achieved using commercial silylamines and standard coupling reagents. Scale up to kilogram quantities is expected to be straightforward. Silylamide trioxanes **4a**, **4b**, and **6** are stable in the absence of solvent for at least 7 days at 60 °C and at least 1 day at 70 °C; less than 2% decomposition was observed by proton NMR spectroscopy. Because these silylamides are C-10 non-acetal trioxanes, they are more hydrolytically stable than the clinically used C-10 acetal trioxane drugs **2c** and **2d**.

### 2.2. Biology

Each trioxane silylamide **4a**, **4b**, and **6** (0.90 mg) was dissolved in 0.11 mL of 7:3 Tween 80:ethanol and then diluted with 1.10 mL of water for oral administration to 5-week old C57BL/6J male mice (from the Jackson Laboratory) weighing 18-19 grams that were infected intraperitoneally on day 0 with the *Plasmodium berghei*, ANKA strain (2 × 10^7^ parasitized erythrocytes) [26]. Each of 3 mice in a group was treated orally 24 hours post-infection with a single dose of 0.20 mL (0.20 mL/1.21 mL × 0.9 mg = 0.15 mg) of diluted compound solution, corresponding to a dose of 8 mg/kg, combined with 24 mg/kg of mefloquine hydrochloride. Determining blood parasitemia levels as well as monitoring the duration of animal survival compared to survival time of animals receiving no drug are both widely accepted as measures of a drug’s efficacy in antimalarial drug development. Three days after infection, an average of 7% blood parasitemia was observed in the control (infected but no drug) group. Animals infected but receiving no drug died on an average of 15 days post infection. A widely accepted yardstick of cure (*i.e.*, 100% efficacy) is survival of animals to at least day 30 post infection, with no detectable malaria parasites in the animal’s blood at that time. Average survival results through day 57 (when the experiment was stopped) are summarized in Table 1.

**Table 1:** Antimalarial Efficacy Using a Single Oral Dose of Trioxane (8 mg/kg) Combined with Mefloquine Hydrochloride (24 mg/kg) in *Plasmodium berghei*-infeced Mice.

Trioxane	Average Survival (days) after Infection	% Suppression of Parasitemia (on day 3 post infection)
**2c**	23	> 99.5
**4a**	> 57	> 99.5
**4b**	44^a^	> 99.5
**6**	> 57	> 99.5

^a^ One mouse died on day 17; the other two mice in this group showed no parasitemia on day 57.

It is clear from Table 1 that both silylamides **4a** and **6** administered as a single oral dose of 8 mg/kg plus mefloquine hydrochloride (24 mg/kg) cured the malaria-infected mice; on day 57 (when the experiment was terminated), no parasites were detected in the blood of the surviving mice. At a single oral dose of 22 mg/kg, mefloquine hydrochloride alone was not curative. In an ACT control experiment, the popular trioxane drug artemether (**2c**) combined with mefloquine hydrochloride prolonged mouse average survival until only day 23 (Table 1). Neither overt toxicity nor behavioral change attributable to trioxane drug administration was observed in any of the malaria-infected animals cured by trioxane **4a** or **6** plus mefloquine hydrochloride combination. By the end of the experiment, all the mice receiving the silylamides **4a** and **6** had gained as much weight (8–10 g) as had the mice in an uninfected control group: this weight gain result is a strong indication of the apparent safety of these silylamides under conditions in which they are curative.

## 3. Experimental

High-pressure liquid chromatography (HPLC) was performed on a Rainin HPLX system equipped with two 25-mL pump heads and a Rainin Dynamax UV-C dualbeam variable wavelength detector set at 254 using a Phenomenex Luna 5 μ C18 250 × 10 mm column. The purity of analogs **4a**, **4b**, and **6** was ≥ 98% based on HPLC analysis. Nuclear magnetic resonance spectra were recorded on a Bruker 400 MHz spectrometer with chloroform as the reference. Infrared spectra were obtained using a Perkin-Elmer Series FT-IR instrument. Optical rotation measurements were taken on a Jasco P-1010 polarimeter. Mass spectroscopy data were obtained using a VG-70S magnetic sector mass spectrometer.

*Synthesis of monomer LW-ART-EtC(O)-NHCH_2_SiMe_3_* (**4a**). Monomer acid ART-EtC(O)-OH (**3** [26], 0.60 g, 1.8 mmol), 1-(3-dimethylaminopropyl)-3-ethylcarbodiimide hydrochloride (EDC, 0.55 g, 2.9 mmol), and hydroxybenzotriazole (HOBt, 0.30 g, 2.2 mmol) were charged into a flame-dried 100 mL round bottom flask at room temperature. Dichloromethane (37 mL) was then added and the mixture was stirred for an hour at which time, (trimethylsilyl)methylamine (0.48 mL, 3.6 mmol) was added by syringe. The reaction was allowed to stir at room temperature for 3 hours. It was then quenched with 1N HCl, extracted with dichloromethane (3 × 5 mL), washed with brine, dried over magnesium sulfate and evaporated. The crude product was purified by preparative thin layer chromatography (silica gel, 40% EtOAc/Hexanes) to afford LW-ART-EtC(O)-NHCH_2_SiMe_3_ (**4a**) as an amorphous, white solid (0.72 g, 1.7 mmol, 94%): IR (thin film) 3295, 2951, 2876, 1743, 1637, 1547, 1451, 1376, 1279, 1249, 1189, 1126, 1094, 1057, 1011, 946, 911, 853 cm^-1^; ^1^H-NMR (400 MHz, CDCl_3_) δ 5.67 (br. s, 1H), 5.26 (s, 1H), 4.04 (m, 1H), 2.74-2.66 (m, 3H), 2.44 (m, 1H), 2.33-2.21 (m, 2H), 2.01-1.74 (m, 5H), 1.63-1.51 (m, 2H), 1.46-1.17 (m, 7H, including singlet at 1.36), 0.95-0.91 (m, 4H), 0.84 (d, 3H, *J*=7.6 Hz), 0.03 (s, 9H); ^13^C-NMR (100 MHz, CDCl_3_) δ 173.20, 103.36, 88.74, 81.13, 75.98, 52.42, 44.45, 37.38, 36.50, 34.55, 34.43, 30.14, 29.78, 26.17, 25.26, 24.85, 24.63, 20.20, 13.15, -2.63; [α]_D_^23^= +61 (c=0.23, CHCl_3_); HRMS (FAB) m/z calcd for C_22_H_39_NO_5_Si (M)^+^ 425.2598, found 425.2584. 

*Synthesis of monomer LW-ART-EtC(O)-NH(CH_2_)_3_SiMe_3_* (**4b**). Monomer acid ART-EtC(O)-OH (**3** [26], 30 mg, 0.088 mmol), EDC (27 mg, 0.14 mmol), and HOBt (15 mg, 0.11 mmol) were charged into a flame-dried 5 mL round bottom flask at room temperature. Dichloromethane (2.5 mL) was then added and the mixture was stirred for an hour at which time, 3-aminopropyltrimethylsilane (24 mg, 0.18 mmol) was added by syringe. The reaction was allowed to stir at room temperature for 3 hours. It was then quenched with 1N HCl, extracted with dichloromethane (3 × 5 mL), washed with brine, dried over magnesium sulfate and evaporated. The crude product was purified by preparative thin layer chromatography (silica gel, 40% EtOAc/Hexanes) to afford LW-ART-EtC(O)-NH(CH_2_)_3_SiMe_3_ (**4b**) as an amorphous, white solid (42 mg, 0.088 mmol, 100%): IR (thin film) 3304, 2951, 2875, 1644, 1547, 1452, 1376, 1248, 1222, 1188, 1126, 1096, 1057, 1012, 938, 912, 862, 837, 753 cm^-1^; ^1^H-NMR (400 MHz, CDCl_3_) δ 5.82 (br. s, 1H), 5.27 (s, 1H), 4.03 (m, 1H), 3.18 (m, 2H), 2.70 (m, 1H), 2.46-2.39 (m, 1H), 2.34-2.21 (m, 2H), 2.01-1.76 (m, 5H), 1.64-1.18 (m, 11H, including singlet at 1.37), 0.95-0.92 (m, 4H), 0.85 (d, 3H, *J*=7.6 Hz), 0.45 (m, 2H), -0.04 (s, 9H); ^13^C-NMR (100 MHz, CDCl_3_) δ 172.91, 103.37, 88.71, 81.16, 76.10, 52.43, 44.47, 42.72, 37.39, 36.52, 34.72, 34.44, 30.17, 26.17, 25.02, 24.86, 24.64, 24.17, 20.21, 13.84, 13.19, -1.76; [α]_D_^23^= +64 (c=0.24, CHCl_3_); HRMS (FAB) m/z calcd for C_24_H_44_NO_5_Si (M+H)^+^ 454.2989, found 454.2980. 

*Synthesis of dimer BTM-isobu-C(O)NHCH_2_SiMe_3_* (**6**). Carboxylic acid dimer **5** (15 mg, 0.03 mmol), EDC (4.4 mg, 0.03 mmol, 1.1 eq) and HOBt (3.8 mg, 0.03 mmol, 1.1 eq) were charged into a flame-dried 5 mL round bottom flask at room temperature. Dichloromethane (1 mL) and (trimethylsilyl)methylamine (4 mL, 0.03 mmol, 1.1 eq) were added, and the reaction mixture was stirred at rt for 2 hours. The reaction mixture was further diluted with CH_2_Cl_2_, and the organic layer was washed with water and sat. aq. NaCl, extracted, dried on MgSO_4_, filtered, and the solvent was removed under reduced pressure. The residue was purified directly on silica. Isocratic elution (25% ethyl acetate in hexanes) afforded the desired product as an amorphous, colorless solid: (17.8 mg, 0.03mmol, 100%): IR (thin film) 3315, 2950, 2875, 1716, 1647, 1540, 1456, 1375, 1249, 1205, 1124, 1093, 1052, 1011, 939, 856; ^1^H-NMR (400 MHz, CDCl_3_) d 5.97 (t, 1 H, *J* = 5.08 Hz), 5.26 (s, 1 H), 5.24 (s, 1 H), 4.09 (m, 1 H), 3.98 (m, 1 H), 2.82 (dd, 1 H, *J* = 5.94, 15.28), 2.72 (sextet, 2 H, *J* = 6.67), 2.66 (dd, 1 H, *J* = 5.05, 15.16), 2.58 (m, 1 H), 2.32 (td, 2 H, *J* = 3.71, 13.96), 2.05 (m, 4 H), 1.86 (m, 4 H), 1.74 (m, 4 H), 1.63 (m, 6 H), 1.51 (m, 6 H), 1.39 (m, 9 H, including singlets at 1.40 and 1.37), 1.25 (m, 6 H), 0.94 (m, 6 H), 0.84 (m, 4 H), 0.07 (s, 9 H); ^13^C-NMR (100 MHz, CDCl_3_) d175.87, 103.46,103.44, 101.08, 88.38, 88.29, 81.19, 81.01, 75.87, 74.26, 52.56, 52.54, 44.76, 44.69, 37.33, 37.26, 36.62, 36.51, 34.51, 29.79, 29.77, 29.71, 26.19, 26.11, 24.73, 24.66, 24.59, 24.56, 20.22, 13.24, -2.42; [a]_D_^23^=+59 (c=0.69, CHCl_3_); HRMS (FAB) m/z calcd for C_38_H_63_NO_9_Si (M+H) 706.4348, found 706.4350.

## 4. Conclusions

In conclusion, a single-digit oral dose of trioxane monomer silylamide **4a** or of trioxane dimer silylamide **6**, combined with mefloquine hydrochloride, is considerably more antimalarially efficacious than the popular ACT trioxane drug artemether (**2c**) combined with mefloquine hydrochloride [17,29,30,31,32]. Complete cure of malaria-infected mice was achieved with silylamides **4a** and **6**, which appear to be safe at the curative dose. Preclinical drug development is continuing, including trioxane lead optimization and use of other amine combination partners.
